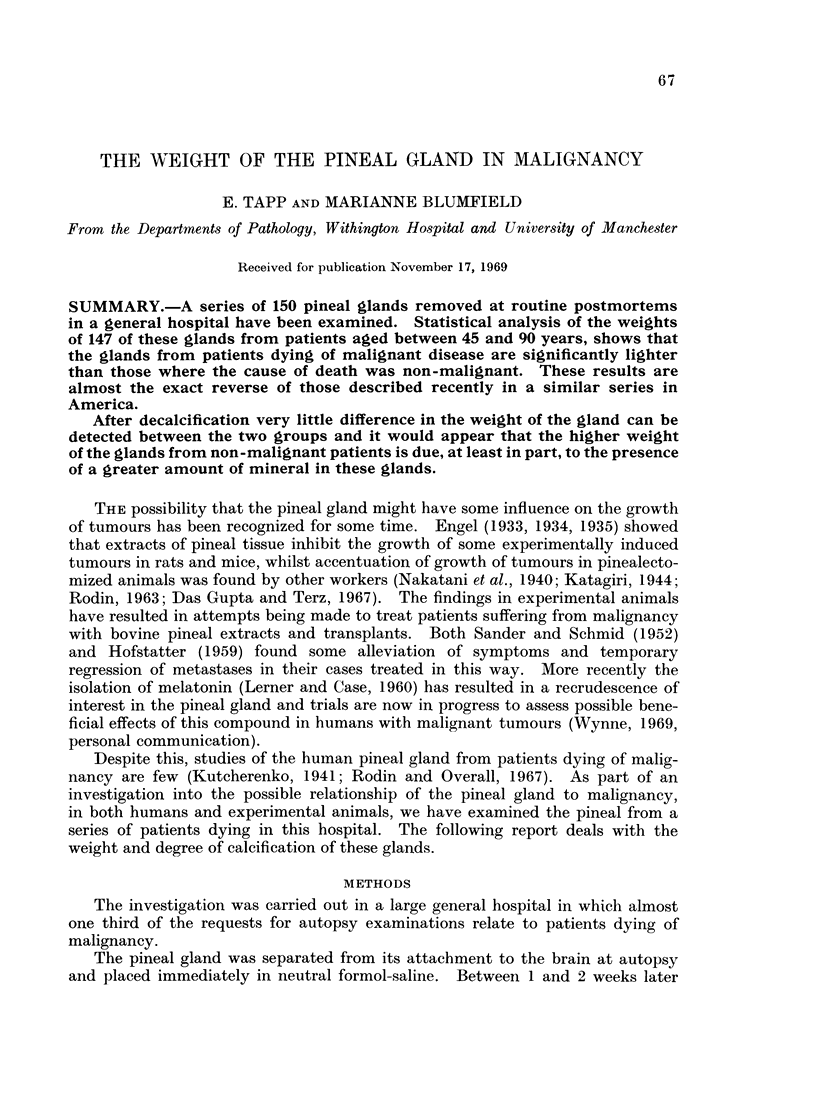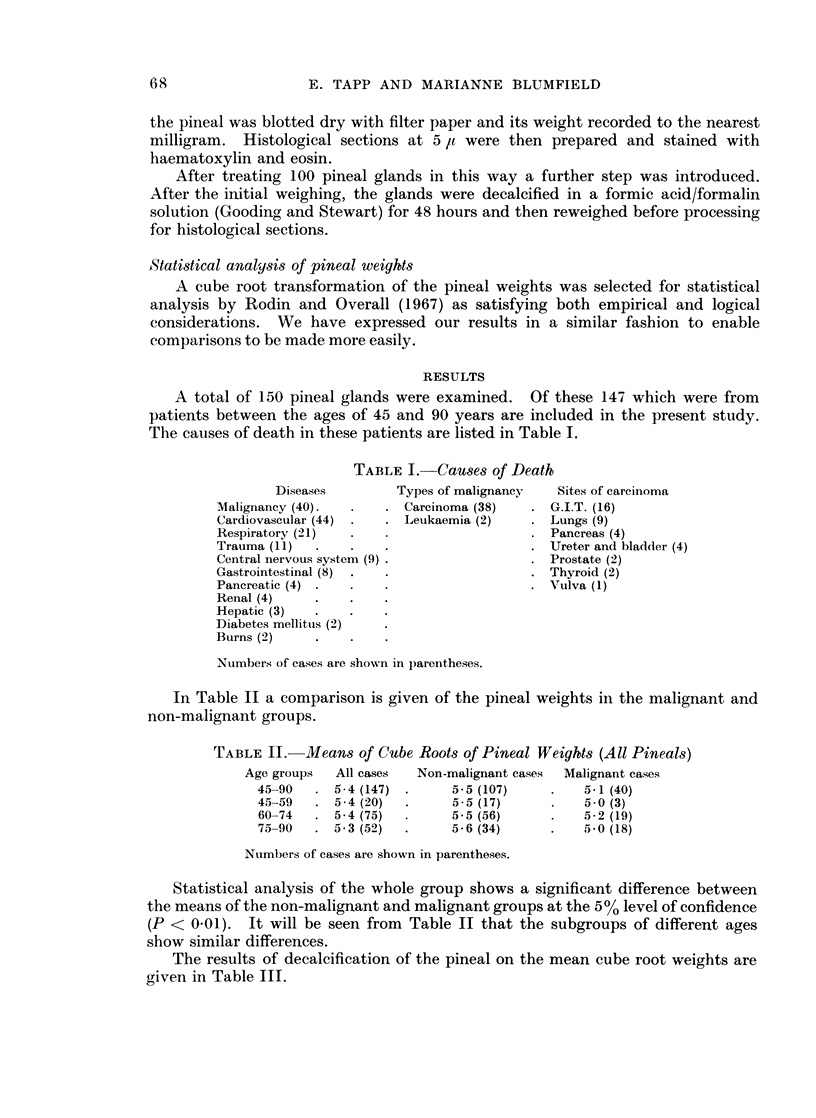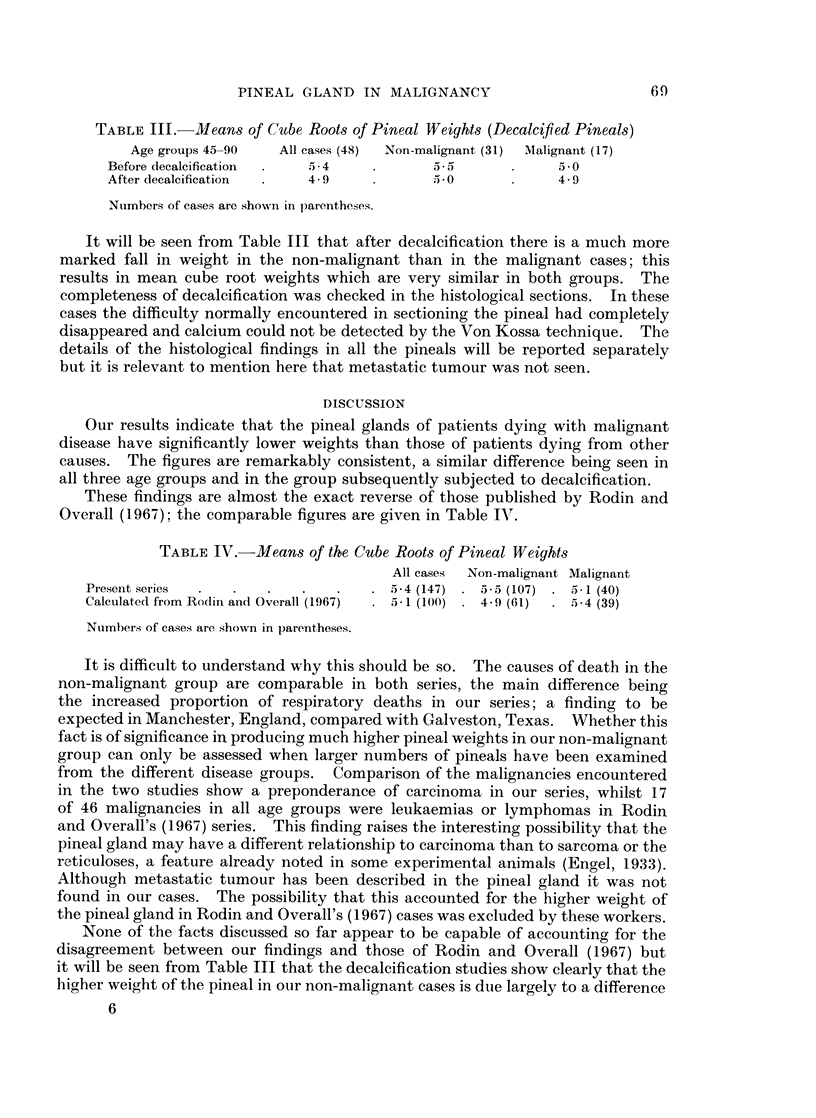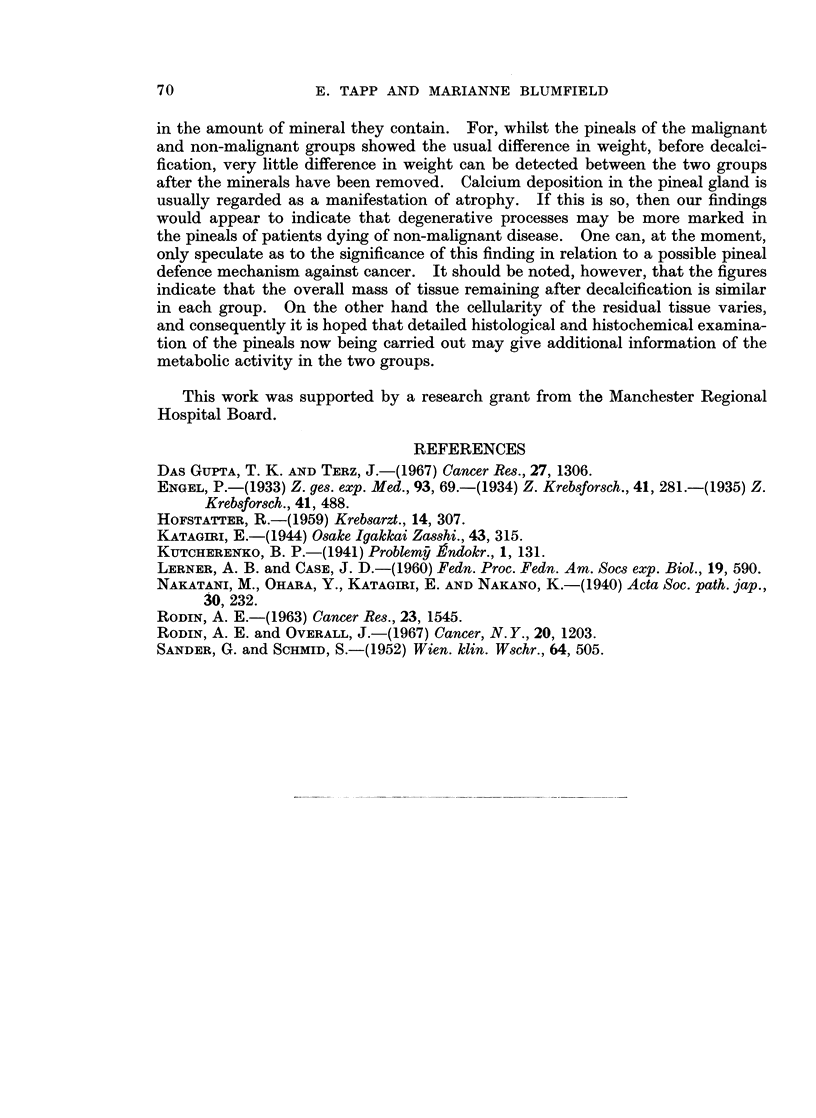# The Weight of the Pineal Gland in Malignancy

**DOI:** 10.1038/bjc.1970.9

**Published:** 1970-03

**Authors:** E. Tapp, Marianne Blumfield

## Abstract

A series of 150 pineal glands removed at routine postmortems in a general hospital have been examined. Statistical analysis of the weights of 147 of these glands from patients aged between 45 and 90 years, shows that the glands from patients dying of malignant disease are significantly lighter than those where the cause of death was non-malignant. These results are almost the exact reverse of those described recently in a similar series in America.

After decalcification very little difference in the weight of the gland can be detected between the two groups and it would appear that the higher weight of the glands from non-malignant patients is due, at least in part, to the presence of a greater amount of mineral in these glands.


### THE WEIGHT OF THE PINEAL GLAND IN MALIGNANCY

E. TAPP AND MARIANNE BLUMFIELD

*From the Departments of Pathology, Withington Hospital and University of Manchester*

Received for publication November 17, 1969

**SUMMARY.—A series of 150 pineal glands removed at routine postmortems in a general hospital have been examined. Statistical analysis of the weights of 147 of these glands from patients aged between 45 and 90 years, shows that the glands from patients dying of malignant disease are significantly lighter than those where the cause of death was non-malignant. These results are almost the exact reverse of those described recently in a similar series in America.**

**After decalcification very little difference in the weight of the gland can be detected between the two groups and it would appear that the higher weight of the glands from non-malignant patients is due, at least in part, to the presence of a greater amount of mineral in these glands.**

THE possibility that the pineal gland might have some influence on the growth of tumours has been recognized for some time. Engel (1933, 1934, 1935) showed that extracts of pineal tissue inhibit the growth of some experimentally induced tumours in rats and mice, whilst accentuation of growth of tumours in pinealectomized animals was found by other workers (Nakatani *et al.*, 1940; Katagiri, 1944; Rodin, 1963; Das Gupta and Terz, 1967). The findings in experimental animals have resulted in attempts being made to treat patients suffering from malignancy with bovine pineal extracts and transplants. Both Sander and Schmid (1952) and Hofstatter (1959) found some alleviation of symptoms and temporary regression of metastases in their cases treated in this way. More recently the isolation of melatonin (Lerner and Case, 1960) has resulted in a recrudescence of interest in the pineal gland and trials are now in progress to assess possible beneficial effects of this compound in humans with malignant tumours (Wynne, 1969, personal communication).

Despite this, studies of the human pineal gland from patients dying of malignancy are few (Kutcherenko, 1941; Rodin and Overall, 1967). As part of an investigation into the possible relationship of the pineal gland to malignancy, in both humans and experimental animals, we have examined the pineal from a series of patients dying in this hospital. The following report deals with the weight and degree of calcification of these glands.

### METHODS

The investigation was carried out in a large general hospital in which almost one third of the requests for autopsy examinations relate to patients dying of malignancy.

The pineal gland was separated from its attachment to the brain at autopsy and placed immediately in neutral formol-saline. Between 1 and 2 weeks later the pineal was blotted dry with filter paper and its weight recorded to the nearest milligram. Histological sections at 5 $\mu$ were then prepared and stained with haematoxylin and eosin.

After treating 100 pineal glands in this way a further step was introduced. After the initial weighing, the glands were decalcified in a formic acid/formalin solution (Gooding and Stewart) for 48 hours and then reweighed before processing for histological sections.

*Statistical analysis of pineal weights*

A cube root transformation of the pineal weights was selected for statistical analysis by Rodin and Overall (1967) as satisfying both empirical and logical considerations. We have expressed our results in a similar fashion to enable comparisons to be made more easily.

### RESULTS

A total of 150 pineal glands were examined. Of these 147 which were from patients between the ages of 45 and 90 years are included in the present study. The causes of death in these patients are listed in Table I.

TABLE I.—*Causes of Death*

| Diseases | Types of malignancy | Sites of carcinoma |
|---|---|---|
| Malignancy (40) | Carcinoma (38) | G.I.T. (16) |
| Cardiovascular (44) | Leukaemia (2) | Lungs (9) |
| Respiratory (21) | | Pancreas (4) |
| Trauma (11) | | Ureter and bladder (4) |
| Central nervous system (9) | | Prostate (2) |
| Gastrointestinal (8) | | Thyroid (2) |
| Pancreatic (4) | | Vulva (1) |
| Renal (4) | | |
| Hepatic (3) | | |
| Diabetes mellitus (2) | | |
| Burns (2) | | |

Numbers of cases are shown in parentheses.

In Table II a comparison is given of the pineal weights in the malignant and non-malignant groups.

TABLE II.—*Means of Cube Roots of Pineal Weights* (*All Pineals*)

| Age groups | All cases | Non-malignant cases | Malignant cases |
|---|---|---|---|
| 45–90 | 5·4 (147) | 5·5 (107) | 5·1 (40) |
| 45–59 | 5·4 (20) | 5·5 (17) | 5·0 (3) |
| 60–74 | 5·4 (75) | 5·5 (56) | 5·2 (19) |
| 75–90 | 5·3 (52) | 5·6 (34) | 5·0 (18) |

Numbers of cases are shown in parentheses.

Statistical analysis of the whole group shows a significant difference between the means of the non-malignant and malignant groups at the 5% level of confidence ($P < 0{\cdot}01$). It will be seen from Table II that the subgroups of different ages show similar differences.

The results of decalcification of the pineal on the mean cube root weights are given in Table III.

TABLE III.—*Means of Cube Roots of Pineal Weights (Decalcified Pineals)*

| Age groups 45–90 | All cases (48) | Non-malignant (31) | Malignant (17) |
|---|---|---|---|
| Before decalcification | 5·4 | 5·5 | 5·0 |
| After decalcification | 4·9 | 5·0 | 4·9 |

Numbers of cases are shown in parentheses.

It will be seen from Table III that after decalcification there is a much more marked fall in weight in the non-malignant than in the malignant cases; this results in mean cube root weights which are very similar in both groups. The completeness of decalcification was checked in the histological sections. In these cases the difficulty normally encountered in sectioning the pineal had completely disappeared and calcium could not be detected by the Von Kossa technique. The details of the histological findings in all the pineals will be reported separately but it is relevant to mention here that metastatic tumour was not seen.

### DISCUSSION

Our results indicate that the pineal glands of patients dying with malignant disease have significantly lower weights than those of patients dying from other causes. The figures are remarkably consistent, a similar difference being seen in all three age groups and in the group subsequently subjected to decalcification.

These findings are almost the exact reverse of those published by Rodin and Overall (1967); the comparable figures are given in Table IV.

TABLE IV.—*Means of the Cube Roots of Pineal Weights*

| | All cases | Non-malignant | Malignant |
|---|---|---|---|
| Present series | 5·4 (147) | 5·5 (107) | 5·1 (40) |
| Calculated from Rodin and Overall (1967) | 5·1 (100) | 4·9 (61) | 5·4 (39) |

Numbers of cases are shown in parentheses.

It is difficult to understand why this should be so. The causes of death in the non-malignant group are comparable in both series, the main difference being the increased proportion of respiratory deaths in our series; a finding to be expected in Manchester, England, compared with Galveston, Texas. Whether this fact is of significance in producing much higher pineal weights in our non-malignant group can only be assessed when larger numbers of pineals have been examined from the different disease groups. Comparison of the malignancies encountered in the two studies show a preponderance of carcinoma in our series, whilst 17 of 46 malignancies in all age groups were leukaemias or lymphomas in Rodin and Overall's (1967) series. This finding raises the interesting possibility that the pineal gland may have a different relationship to carcinoma than to sarcoma or the reticuloses, a feature already noted in some experimental animals (Engel, 1933). Although metastatic tumour has been described in the pineal gland it was not found in our cases. The possibility that this accounted for the higher weight of the pineal gland in Rodin and Overall's (1967) cases was excluded by these workers.

None of the facts discussed so far appear to be capable of accounting for the disagreement between our findings and those of Rodin and Overall (1967) but it will be seen from Table III that the decalcification studies show clearly that the higher weight of the pineal in our non-malignant cases is due largely to a difference in the amount of mineral they contain. For, whilst the pineals of the malignant and non-malignant groups showed the usual difference in weight, before decalcification, very little difference in weight can be detected between the two groups after the minerals have been removed. Calcium deposition in the pineal gland is usually regarded as a manifestation of atrophy. If this is so, then our findings would appear to indicate that degenerative processes may be more marked in the pineals of patients dying of non-malignant disease. One can, at the moment, only speculate as to the significance of this finding in relation to a possible pineal defence mechanism against cancer. It should be noted, however, that the figures indicate that the overall mass of tissue remaining after decalcification is similar in each group. On the other hand the cellularity of the residual tissue varies, and consequently it is hoped that detailed histological and histochemical examination of the pineals now being carried out may give additional information of the metabolic activity in the two groups.

This work was supported by a research grant from the Manchester Regional Hospital Board.

### REFERENCES

Das Gupta, T. K. and Terz, J.—(1967) *Cancer Res.*, **27**, 1306.
Engel, P.—(1933) *Z. ges. exp. Med.*, **93**, 69.—(1934) *Z. Krebsforsch.*, **41**, 281.—(1935) *Z. Krebsforsch.*, **41**, 488.
Hofstatter, R.—(1959) *Krebsarzt.*, **14**, 307.
Katagiri, E.—(1944) *Osake Igakkai Zasshi.*, **43**, 315.
Kutcherenko, B. P.—(1941) *Problemÿ Éndokr.*, **1**, 131.
Lerner, A. B. and Case, J. D.—(1960) *Fedn. Proc. Fedn. Am. Socs exp. Biol.*, **19**, 590.
Nakatani, M., Ohara, Y., Katagiri, E. and Nakano, K.—(1940) *Acta Soc. path. jap.*, **30**, 232.
Rodin, A. E.—(1963) *Cancer Res.*, **23**, 1545.
Rodin, A. E. and Overall, J.—(1967) *Cancer, N.Y.*, **20**, 1203.
Sander, G. and Schmid, S.—(1952) *Wien. klin. Wschr.*, **64**, 505.